# Mechanisms of Hypomagnetic Field Biological Effects: Quantum Rotator Versus Radical Pair

**DOI:** 10.3390/ijms27198855

**Published:** 2026-10-04

**Authors:** Vladimir Binhi, Igor Usovik, Ivan Koshel

**Affiliations:** 1 Federal State Budgetary Institution “Federal Scientific and Clinical Center for Space Medicine and Biology” of the Federal Medical Biological Agency, Suschevskiy Val 24, 127018 Moscow, Russia; usovikiv@mail.ru (I.U.);; 2 Faculty of Biology, Lomonosov Moscow State University, Leninskie Gory 1/12, 119234 Moscow, Russia; 3 Prokhorov General Physics Institute of the Russian Academy of Sciences, Vavilov St. 38, 119991 Moscow, Russia

**Keywords:** weak magnetic field, nanorotator, magnetic biological effects, open quantum system, Liouville–von Neumann equation, quantum coherence, enzyme, biopolymerization, translation, protein

## Abstract

The radical pair mechanism (RPM) struggles to explain the biological effects of weak magnetic fields due to rapid spin decoherence. To address this, we propose a mechanism based on the quantum dynamics of a sub-nanometric (<0.5 nm) molecular rotator modeling an amino acid residue within a biosynthesizing enzyme. Using the Liouville–von Neumann equation with chemical kinetics and phenomenological decoherence, we show that MF effects arise from modulation of the rotator’s quantum interference pattern rather than from spin prohibition. Our results demonstrate that effects reaching several tens of percent occur in very weak magnetic fields (below 50 μT), are relatively robust to decoherence, and that while temperature promotes decoherence, it also enables the formation of a magnetic field-sensitive interference pattern. Under realistic parameters, the angular distribution of the rotator’s probability density rotates by several degrees in the geomagnetic field. The rotator mechanism implies that evolution has adapted organisms to use the geomagnetic field for nearly error-free biosynthesis; under hypomagnetic fields, biosynthetic errors increase. Unlike the RPM, this mechanism achieves high sensitivity by operating via magnetic field-induced phase changes rather than magnetic moment energy, thereby circumventing the standard quantum limit. Although tentative and lacking direct experimental evidence, the quantum rotator mechanism provides a biophysically transparent interpretation of hypomagnetic field effects, magnetic storms, and animal magnetic navigation, opening new avenues for research and various applications.

## 1. Introduction

There is extensive literature on the biological effects of weak magnetic fields [1,2,3]. It has been reliably established that even small changes in the magnetic field (MF) in an organisms’ environment often cause a wide array of effects [4,5]. However, the mechanism underlying the biological response to MF is not yet fully understood. Such a mechanism must ensure a noticeable change in the probability of an individual chemical reaction event in an MF on the order of the geomagnetic field (geoMF, about 50 μT), particularly upon its significant attenuation to hypomagnetic levels. Many cell cultures and plants that respond to MF do not contain magnetic nanoparticles whose presence could explain the observations. Therefore, elucidating a common *molecular* mechanism of the biological effects of MF continues to be a central challenge.

The primary molecular mechanism cannot be understood other than as the action of MF on magnetic moments, specifically on the quantum dynamics of electrons. Less frequently discussed is the possible role of nuclear magnetic moments [6]. Due to changes in the quantum states of electrons, initial magnetic signals arise, which are then converted into the biochemical and biological responses observed in experiments.

An electron possesses a spin magnetic moment as well as a magnetic moment associated with its spatial motion along a closed trajectory—the orbital magnetic moment. According to the types of electronic magnetic moments, there exist two initial mechanisms of the molecular action of an MF on electrons. These are the well-known spin-chemical radical pair mechanism (RPM) [7] and the little-known quantum rotator mechanism [8] involving the orbital magnetic moment.

In searching for and identifying the true molecular mechanism, a special role is played by the use of a hypomagnetic field (hypoMF)—i.e., an MF that is orders of magnitude weaker than the geoMF. In a hypoMF, the quantum dynamics of magnetic moments slows down, regardless of their nature. This greatly facilitates the interpretation of experimental data. In the present work, we consider the two above-mentioned primary mechanisms of the magnetic biological response under a thousandfold reduction in the MF from the geoMF level.

After more than half a century of study and development, the RPM has not fully explained the observed effects of weak MFs on organisms [9]. Therefore, the quantum rotator mechanism is now gaining a chance for revival [10] and re-evaluation. The advantages of this mechanism are best revealed when compared to the RPM.

The RPM is the leading theory today for explaining the biological effects of low-intensity MFs [11,12,13]. According to the angular momentum conservation law and the Pauli exclusion principle, a chemical reaction occurs only if the electron spins of the radicals acquire a specific mutual orientation. In the quantum approach, the spin state of a radical pair is described by a superposition of singlet-triplet states and their corresponding density matrix ρ. The reaction is then considered to proceed from a specific state, for example, the singlet state.

The master equation commonly used in the RPM for analyzing spin dynamics has the form of the Liouville–von Neumann equation, supplemented by superoperators of chemical kinetics K and relaxation R [14,15],

(1) $$\partial_{t}\;\rho =-i\;\left[H,\rho \right]+K\left(\rho \right)+R\left(\rho \right),$$

where $\partial_{t}$ denotes the partial derivative with respect to time and H is the spin Hamiltonian of the system. In Ref. [16], an investigation of this equation with various K but in the absence of R showed that its solutions successfully describe some characteristics of the effects observed in the magnetic orientation of magnetosensitive animals. Some migratory birds do not react to the reversal of the MF direction, which is a distinctive feature of quantum-mechanical RPM models.

A key example is RPM applied to flavin–tryptophan radical pairs in retinal cryptochromes under blue light in migrating birds, sea turtles, and bees [12]. Evidence for cryptochrome involvement includes: in vitro MF sensitivity (though at fields orders of magnitude above geoMF), spectral dependence matching flavin absorption, and ordered orientation enabling directional MF response [17].

However, the striking discrepancy remains unexplained: magnetic *biological* effects in vivo often reach over ten percent, while spin-*chemical* RPM effects with cryptochromes in vitro are typically less than a tenth of a percent. Theoretical calculations within the RPM framework, accounting for decoherence R(ρ), also predict effects that are too small to explain the biological observations [9].

RPM effects arise when the radical pair’s quantum state is well-protected from environmental perturbations, which is equivalent to requiring a sufficiently long spin coherence relaxation time τ. An analytical solution of the Liouville–von Neumann equation

(2) $$\partial_{t}\;\rho =-i\;\left[H,\rho \right]-k\rho -g\left[\rho -\mathrm{tr}\left(\rho \right)\bar{\rho }\right]$$

for an idealized system of two electrons and one nucleus with minimal interactions, subject to a static MF, spin decoherence, and chemical kinetics (with dimensionless rate *k*), was obtained in [18]. The relaxation superoperator used was $R\left(\rho \right)\equiv -g[\rho -\mathrm{tr}\left(\rho \right)\bar{\rho }]$, where *g* is the relaxation rate and $\bar{\rho }$ is the density matrix at thermal equilibrium. The case of equal relative rates of chemical kinetics for the singlet and triplet channels $k_{S}=k_{T}=k$ was considered, so that the Haberkorn superoperator $K\left(\rho \right)\equiv -\frac{1}{2}\left\{k_{S}P_{S}+k_{T}P_{T},\rho \right\}$, where $P_{S}$ and $P_{T}$ are the projectors onto the singlet and triplet state subspaces, and {,} denotes the anticommutator, reduced to K(ρ)=−kρ.

The indicated solution to (2) allows one to calculate the relative magnetic RPM effect

(3) $$M\left(H,k,g\right)=1-\frac{\int_{0}^{\infty }F\left(t,H,k,g\right)\;dt}{\int_{0}^{\infty }F\left(t,0,k,g\right)\;dt},$$

where *F* is the time evolution of the singlet state population of the pair and *H* is the magnitude of the MF. An analysis of this solution showed that the magnitude of the RPM effect, within the parameter range relevant to magnetobiology, is described to a sufficiently good approximation by relation

(4) $$M_{\mathrm{RPM}}\approx \frac{1}{5}{\left(\gamma H\tau \right)}^{2}\kappa \tau ,$$

where γ is the electron spin gyromagnetic ratio, τ∝1/g is the decoherence time, and κ is the kinetic rate. Calculations using this formula agree with the experimental in vitro data [19,20,21,22,23] on flavin–adenine and flavin–tryptophan radical pairs in photolyases and cryptochromes. The agreement is achieved with decoherence time τ ranging from 3 to 30 nanoseconds and a typical value of κ∼1 μs^−1^ for magnetosensitive radical pairs in these proteins.

The magnitude of RPM effect (4) becomes significant when the equality

(5) γHτ∼1+κτ

holds. This constraint modifies the relation γHτ∼1, which follows from the energy–time uncertainty relation [24], for the case when chemical kinetics is included. Given the values of κ and τ indicated above, a radical pair exhibits a noticeable response to an MF only at magnitudes on the order of 1 mT. For weak MFs, such as the geoMF (0.05 mT), the RPM signal would be less than 0.1%.

This sensitivity estimate for RPM models developed in magnetobiology assumes the minimal spin-interaction configuration specified above and decoherence times below 30 ns. While future, more advanced RPM models may lift this constraint, it seems to be quite general for now.

It appears that, remaining within the framework of physics, the smallness of the primary magnetic signal of the RPM cannot be overcome, as follows from constraint (5). However, organisms somehow cope with or circumvent this limitation. This motivates the search for alternative MF detection mechanisms that would be distinct from RPM and rooted in biological processes. Examples are recurring catalytic cycles: DNA/RNA biopolymerization and protein synthesis. In these (quasi)cyclic processes, the error probability grows with chain length and may depend on the MF [25].

In these processes, large molecular fragments act as nanoscopic transient rotators. Even when neutral, they possess large electric dipole moments due to electron cloud displacement from nuclei, meaning separated charge distributions. The rotation of such amino acid residue generates a magnetic moment that couples with the external MF. As theoretically estimated in [8], the thermalization time τ of the rotational state for the residue can reach up to 0.1 s.

For such rotators, the criterion γHτ∼1 retains its significance. Although the gyromagnetic ratio of a rotating molecule is many orders of magnitude smaller than that of an electron, the values of τ are increased by nearly the same number of orders of magnitude compared to the electronic case. Thus, explaining MF effects in organisms warrants considering orbital degrees of freedom of the electron as a potential alternative to spin.

An amino acid residue is much heavier than a single atom. While classical rotational dynamics suffices for a thermalized rotator, the slow thermalization after its formation or release allows quantum effects to emerge—as assumed in the present work.

The quantum rotator mechanism models the rotation of a segment of a protein chain, or an amino acid residue, in the ribosome during translation, i.e., protein synthesis according to the mRNA code. Upon the incorporation of a new residue into the growing protein chain, it transitions from a state bound to tRNA to a state bound to the chain ([26] p. 984). We assume that during this short time interval, the residue resides in the active site of the enzyme in a relatively free cavity, where its interaction with the thermal fluctuations in the environment is weakened. This intermediate state resembles the spin-correlated state of a radical pair in the RPM. The residue retains covalent bonds on both sides (tRNA and the protein chain) and can rotate about the axis formed by these bonds. This rotational degree of freedom eventually thermalizes. However, initially it is shielded from thermal vibrations of the covalent bonds [8]. The vibrations of these bonds do not affect the angular momentum due to the angular momentum conservation law.

It is known that substrate selection in the ribosomal A site proceeds through initial selection and kinetic proofreading [27]. As a distinct level of control associated with tRNA accommodation, the induced conformational change in the peptidyl–transferase center (induced fit PTC) is considered [28]. Peptide bond formation itself is the final chemical reaction. The stage at which the amino acid is oriented is the induced fit PTC. According to [29], the active center can initially retain the amino acid in non-reactive conformers (rotamers), in which its amino group is not directed toward the reaction. Binding of the 3′-end of the tRNA triggers a rearrangement of the center: non-reactive conformers cease to be retained, and the amino acid rotates into the position required for peptide bond formation. Without this rotation, the bond is not formed. We propose that this rotation—the positioning of the substrate within the catalytic center of the PTC at the stage of chemical accommodation—is sensitive to an external MF. In the geoMF, the rotation proceeds normally and errors are rare. In a hypoMF, it could be slowed down or distorted. Then, correct tRNAs would fail to rotate in time, whereas incorrect ones would be less likely to be rejected promptly, increasing the probability of misincorporation.

In an MF, such a molecular rotator rotates according to quantum laws with a speed proportional to the field, as will become clear from what follows. During its lifetime in a non-thermalized quantum state, the nanorotator manages to rotate by several degrees. We assume that the functioning of the enzyme is sensitive to such rotations and that, in the geoMF, the rotation of the residue is evolutionarily fixed and represents the norm. However, in a hypoMF, quantum rotation is absent, the protein is synthesized with an increased error rate, becomes toxic, and induces nonspecific biological reactions.

We emphasize that this involves a single rotational degree of freedom—the rotation of an entire amino acid residue about a single axis. Such degrees of freedom are very few compared to other rotational degrees in a protein, and they exist only briefly. Therefore, standard spectroscopic methods do not detect these transient degrees of freedom. A natural way to detect them is biochemical, particularly using an MF.

The amino-acid rotation accompanying induced fit PTC, as reported by [29], still lacks independent experimental confirmation. Accordingly, the account below of the quantum MF influence on this rotational degree of freedom—while consistent with existing physical knowledge and with numerous observations—remains a well-grounded theoretical construct.

The present work aims to assess the effects arising from the described scenario, in which a biochemical event is activated by the absence of quantum rotation in a hypoMF. It was necessary to (i) calculate the magnitude of the primary magnetic response by solving the Liouville–von Neumann equation with kinetics and decoherence, (ii) estimate the magnetic effect for a specific amino acid residue, (iii) show that, due to interference, the quantum rotator mechanism modifies the fundamental constraint γHτ∼1, yielding, in comparison with the RPM, a minimum detectable field two to three orders of magnitude smaller (1 μT) and a magnetic effect magnitude three orders of magnitude greater under realistic parameter values, and (iv) discuss possible implications for magnetobiological experiments in biology.

In Section 2.1, the dynamics of a transient rotator in an MF are considered; in Section 2.2, quantum rotation; in Section 2.3, magnetic effect; and in Section 2.4, estimation of the gyromagnetic ratio for Asp. Section 3 contains a discussion of various aspects of the rotator mechanism: comparison of the two mechanisms in Section 3.1, experimental distinction from the RPM in Section 3.2, and possible influence on certain proteins in Section 3.3. Methods used are presented in Section 4 and a brief formulation of the conclusions is given in Section 5.

## 2. Results

### 2.1. Nanorotator in a Magnetic Field

As is shown in [18], the formal solution of Equation (2) is

(6) $$\rho \left(t\right)=\theta_{1}U\sigma U^{†}+\theta_{2}\bar{\rho },\;\;\theta_{1}\equiv e^{-(k+g)t},\;\;\theta_{2}\equiv e^{-kt}\left(1-e^{-gt}\right),$$

where U(t)≡exp(−iHt) is the Hamiltonian evolution operator, σ≡ρ(0) is the initial density, and $\bar{\rho }$ is the density of the equilibrium state in the absence of chemical kinetics. When applying this solution to a nanorotator, it is convenient to use a finite-dimensional basis and to represent the equilibrium state as a completely mixed state, with its density matrix taken to be diagonal. Unlike [10], wherein the equilibrium state follows Boltzmann statistics, the present work employs an equilibrium density matrix with equipopulated levels. This idealization affords significant mathematical simplification and an analytical solution for the magnetic effect, while preserving the model’s essential physics.

We use the Hilbert state space of dimension N≡2L+1, where *L* denotes the maximum orbital quantum number (angular momentum), which is sufficiently large to ensure that rotator states with energies up to kT can be populated under thermal perturbations, while states with quantum numbers exceeding *L* have negligible population.

As mentioned above, in analytical models of RPM effects, the chemical reaction is often considered equally effective for singlet and triplet precursors, which reduces the action of the chemical superoperator to the form −kρ. Similarly, in the quantum rotator model, as a rough approximation, we also assume chemical damping of ρ from all states, so that the chemical superoperator takes the same form. Then, solution (6) remains valid, and the chemical reduction in the density matrix is given by $\mathrm{tr}\left(\rho \right)=e^{-kt}$.

The idealization of a molecular rotator is a rigid system of masses and charges rotating about a fixed axis. As obtained in [2], the Hamiltonian of such a rotator in the field of the vector potential (−yH,xH,0)/2 (which corresponds to a constant uniform MF *H* along the *z* axis) takes the form $\Lambda^{2}/2I\equiv {\left(\hbar L-I\gamma H\right)}^{2}/2I$, where

(7) Λ≡ℏL−IγH

is the kinetic (measurable) angular momentum, L≡−i∂/∂φ is the dimensionless operator of the canonical quantized angular momentum, *I* is the moment of inertia, and γ is the orbital gyromagnetic ratio of the molecule. The term in the Hamiltonian proportional to $H^{2}$ is omitted hereafter due to its smallness.

Frequency unit ω≡ℏ/2I is introduced for convenience. This allows for the definition of the following dimensionless values of time *t*, MF *x*, rates k,g, and energy ϵ:

(8) $$t\equiv \omega t^{\prime },\;\;x\equiv \gamma H/\omega =2I\gamma H/\hbar ,\;\;k\equiv \kappa /\omega ,\;\;g\equiv \Gamma /\omega \equiv 1/\omega \tau ,\;\;\epsilon =E/\hbar \omega ,$$

where $t^{\prime }$ is physical time, *x* is proportional to the MF *H*, κ and Γ=1/τ are the physical rates of chemical kinetics and thermal relaxation, τ is the relaxation time of quantum coherence, and *E* is energy. The Hamiltonian (dimensionless) becomes

(9) $$H=L^{2}-xL,$$

while the Schrödinger equation takes the dimensionless form $\partial_{t}\Psi \left(\varphi \right)=-i\;H\Psi \left(\varphi \right)$.

In the energy representation, the eigenvectors |n〉 of H correspond to the orthonormal functions of the coordinate representation $\phi_{n}\left(\varphi \right)$ with eigenvalues $\varepsilon_{n}$

(10) $$\phi_{n}\left(\varphi \right)=\frac{1}{\sqrt{2\pi }}e^{i\;n\varphi },\;\varepsilon_{n}=n^{2}-xn,\;n=-L,\ldots ,L$$

where *n* is the magnetic quantum number, *L* is its maximum value defined above, and φ is the angular coordinate.

The condition $\hbar \omega \varepsilon_{n}∼kT$ means the initial thermal population of the eigenstates up to $n∼\sqrt{kT}/\sqrt{\hbar \omega }∼100$. Therefore, we set L=100 below.

### 2.2. Quantum Rotation in a Magnetic Field

To employ Equation (6), one must define the initial density matrix σ≡|ψ〉〈ψ|, where |ψ〉 is the state of the rotator that emerges upon its release and constitutes the initial state for the dynamical problem. We assume that before t=0, the rotator is confined to the fixed angular position φ=0. Note that the rotational state of a fixed rotator is not thermalized: until the rotator is released, this degree of freedom is not dynamical.

A suitable idealization of the bound quantum state used in [10] is an eigenstate of the rotator in an attractive δ-potential, in which the rotator wavefunction is known to be unique [30].

Since it is the only possible state of a fixed rotator, it cannot be mixed. This is analogous to the RPM case, in which the two spins are in a non-thermalized pure singlet state after the formation of a radical pair. We further assume that the release of the rotator occurs much faster than the characteristic time of its subsequent evolution. Upon the fast release of the rotator, its wave function ψ remains unaltered. It becomes a pure superposition of rotational modes (10), $|\psi \rangle =\sum_{n}c_{n}|n\rangle$; the summation here and henceforth runs from −L to *L*. Only after that does the process of decoherence and the transition to a mixed state begin.

In MF, the kinetic angular momentum operator (7) of the rotator Λ≡ℏL−IγH=ℏL−ℏx/2 contains, in addition to the canonical angular momentum ℏL, the electromagnetic contribution ℏx/2. As is clear for a bound, or fixed, state of the rotator, its average kinetic angular momentum 〈ψ|Λ|ψ〉 equals zero, so that the canonical and electromagnetic momenta completely cancel each other out.

After release, the kinetic angular momentum remains zero. Angular momentum is conserved because it commutes with the Hamiltonian, $\left[\Lambda ,H\right]=\left[\hbar L-\hbar x/2,\;L^{2}-xL\right]=0$. This, however, does not exclude quantum rotation, i.e., rotation of the angular probability density distribution of the rotator. The corresponding operator is the probability current density operator $J\propto \psi^{*}L\psi -\psi L\psi^{*}$. It can be shown that in a bound state its mean value is zero, 〈J〉=0, whereas in a free state it is not. This is the paradoxical quantum rotation: the rotator, as an ensemble of masses, does not rotate, but the angle at which it could be detected with the highest probability does rotate. Accordingly, the total magnetic moment γΛ is conserved and has zero mean. However, the MF induces the quantum rotation of the probability density, generating a mean canonical magnetic moment $\mathrm{tr}\left(L\rho \right)=\gamma \hbar x\;e^{-kt}/2$.

The magnetic moment is extremely small due to its proportionality to x≪1 and plays no role in the energetics of rotations. One can say that it is a byproduct. A truly interesting phenomenon is that the change in the wave functions of the rotator in MF produces the quantum rotation. Unlike classical macroscopic rotation, this quantum rotation, arising from eigenstate interference in an MF, is inertialess.

Quantum rotation is clearly seen in the coordinate representation. The density *W* of the probability of finding the rotator at position φ is the “diagonal” element of the density matrix in the coordinate representation,

$$W\left(t,\varphi \right)=\sum_{nm}\rho_{nm}\left(t\right)\;\phi_{n}^{*}\left(\varphi \right)\phi_{m}\left(\varphi \right).$$

From Definitions (6) and (10), the normalization of the density matrix to its chemically reduced value is as follows: $\int_{0}^{2\pi }W\left(\varphi ,t\right)d\varphi =\sum_{nm}\rho_{nm}\delta_{nm}=\mathrm{tr}\left(\rho \right)=e^{-kt}$, where $\delta_{nm}$ is the Kronecker symbol. Substituting ρ and ϕ from these definitions, and taking equilibrium density matrix $\bar{\rho }\equiv 1/\mathrm{tr}1$, or ${\bar{\rho }}_{nm}=\delta_{nm}/N$, we write:

(11) $$W\left(t,\varphi \right)=\frac{1}{2\pi }\left(\theta_{1}s_{1}+\theta_{2}s_{2}\right),$$

where notations for the sums *s* are introduced,

(12) $$s_{1}\equiv \sum_{nm}{\left(U\sigma U^{†}\right)}_{nm}e^{-i\;(n-m)\varphi },\;\;s_{2}\equiv \frac{1}{N}\sum_{nm}\delta_{nm}\;e^{-i\;(n-m)\varphi }=1.$$

To calculate the sum $s_{1}$, let us first find the elements of the matrix $U\sigma U^{†}$, where, as above, we set σ=|ψ〉〈ψ|. Applying the spectral decomposition of the evolution operator $U=\sum_{i}\lambda_{i}\left|i\rangle \langle i\right|$, where |i〉〈i| are the projectors onto the basis states, and $\lambda_{n}\equiv \exp\left(-i\;\varepsilon_{n}t\right)=\exp\left[i\;\left(nx-n^{2}\right)t\right]$ are their eigenvalues, we find

$$U\sigma U^{†}=\sum_{ik}E_{ik}\left|i\rangle \langle i|\psi \rangle \langle \psi |k\rangle \langle k\right|=\sum_{ik}E_{ik}|i\rangle \sigma_{ik}\langle k|,$$

where *E* is the matrix with elements $E_{ik}=\lambda_{i}\lambda_{k}^{*}$. Hence, ${\left(U\sigma U^{†}\right)}_{nm}=\sum_{ik}E_{ik}\langle n|i\rangle \sigma_{ik}\langle k|m\rangle =E_{nm}\sigma_{nm}$. Substituting this into $s_{1}$ in (12) and assuming, for convenience, that the initial state is a superposition of equally populated eigenstates, i.e., $\sigma_{nm}=1/N$, we obtain the sum in the following form

$$s_{1}=\frac{1}{N}\sum_{nm}e^{-i\;(n-m)(xt-\varphi )}e^{-i\;(n^{2}-m^{2})t}.$$

The initial state ψ, taken as a superposition of equally populated eigenstates, differs from the exact solution of the Schrödinger equation for a rotator in an attractive δ-potential with parameter kT, obtained in [10]. It is, however, close to the exact solution. The wave function ψ captures the main features of that solution—its narrowness and height in accordance with kT, its symmetry, and its nearly exponential decay with increasing φ. The ψ we use is a well-justified approximation that yields a simple analytical relationship between the MF and the resulting magnetic effect.

Recall that all sums are taken from −L to L≫1. In this sum, there are terms with m=±n, and extremely rapidly oscillating terms with m≠±n, with about $N^{2}/2$ different frequencies. Therefore, the sum of these mutually compensating terms is very small and can be neglected. Calculating the terms in $s_{1}$ with m=±n, we find

$$s_{1}=1+\frac{\sin[N(xt-\varphi )]}{N\sin(xt-\varphi )}.$$

Hence, substituting θ, $s_{1}$, and $s_{2}$ into (11) we obtain for L≫1

(13) $$W\left(t,x,\varphi \right)=\frac{e^{-kt}}{2\pi }\left[1+e^{-gt}\;\frac{\sin[2L(xt-\varphi )]}{2L\sin(xt-\varphi )}\right].$$

**Figure 1:**
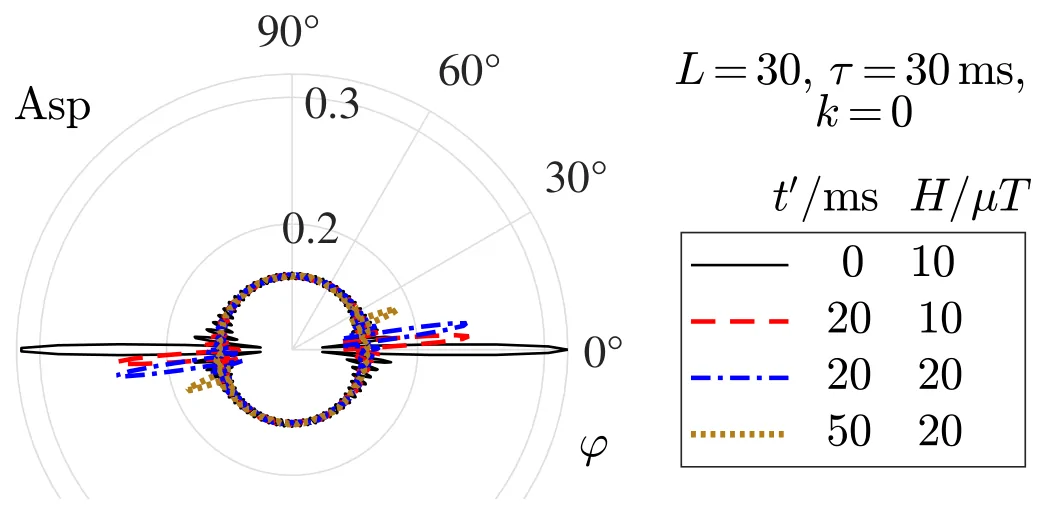
Rotation of the probability density *W* of the rotator, representing the Asp residue, at different values of physical time and MF and in the absence, for clarity, of a chemical reduction. The probability density features a prominent, narrow peak, or spike.

Figure 1 shows this relation in the form of a π-periodic function of the polar angle φ for different values of time and MF, which were converted from the dimensionless parameters *t* and *x* according to (8). Calculations are performed for a nanoscopic rotator in the form of an aspartic acid (Asp) residue. An estimation of parameter values is given in the next section.

It can be seen from the figure that the probability density of the rotator being at the angular position φ is a wave packet with a narrow spike with a maximum height of 1/π against a constant background of slightly less than 1/2π. Its width is 2/L. The nanoscopic nature of the rotator is manifested here in the narrowness of the spike. With good reason, this spike can be called a quantum needle.

As the mass of the rotator increases, the number of populated states increases and their interference produces an increasingly narrow needle. It disappears at the macroscopic limit. At the same time, the constant component tends toward 1/2π. This is how the transition from quantum to classical dynamics occurs here. It can also be said differently: the peak in the wave packet rotates, as follows from the argument xt−φ, with an angular velocity *x*, or γH in physical units. The greater the MF, the faster this quantum rotation. The direction of rotation is determined by the sign of the MF, and its speed is proportional to γ. As the size of the rotator increases, the gyromagnetic ratio decreases and this effect disappears.

The transition from quantum to classical motion also occurs in time: as the rotator thermalizes, coherent rotation is replaced by Brownian rotation. This transition is represented phenomenologically by the coefficient *g*. With time *t* the peak in Figure 1 shifts. Simultaneously its height, due to the factor $e^{-(k+g)t}$ in (13), decreases rapidly. As the angular width of the peak is 2/L, the shift by half the peak width occurs in time t=1/xL. For the magnetic effect to be noticeable, the decrease in height should not be too fast, i.e., (k+g)t<1, or xL>k+g. In terms of physical variables (8), this leads to a modified observability criterion,

(14) γHτL>1+κτ.

This generalizes the fundamental RPM relation (5) to the case of about 2L interfering states, whose “measurement,” in the form of a chemical reaction act, occurs simultaneously. The critical MF decreases by approximately a factor of *L*~100 compared to the case of a measurement in a few-level spin system. In fact, the quantum rotator, undergoing a reaction with an active neighbor only in a certain narrow angular sector, is a kind of interference amplifier of the weak magnetic signal.

### 2.3. Magnetic Effect Arising from Quantum Rotation

Let us recall that in this scenario, rotation in the geoMF is the evolutionary norm. This rotation displaces the peak away from its initial position, where the probability of an erroneous biochemical reaction is increased. An adverse biological effect occurs if such rotation is absent, i.e., with a decrease in the MF to hypomagnetic levels.

To estimate the resulting magnetic effect, one must calculate the time evolution of q≡tr[σρ(t)], i.e., the population of the state coinciding with the initial one and contributing to the occurrence of biopolymerization errors, and then average it over time. It is important that there is no classical “return” of the rotator to the initial state. This is a quantum projection of one state onto another, which has no classical analogy. Here, we take into account that the projector onto the initial state coincides with density matrix σ of this state.

Using solution (6), we write the population in the following form:

(15) $$q\left(t,x,k,g,L\right)=\theta_{1}\;\mathrm{tr}\left(\sigma U\sigma U^{†}\right)+\theta_{2}\;\mathrm{tr}\left(\sigma \bar{\rho }\right).$$

The dependence on MF *x* is contained in the evolution operator *U*. First, we need to find $\mathrm{tr}\left(\sigma U\sigma U^{†}\right)$. Again, keeping in mind that σ≡|ψ〉〈ψ|, we write $\mathrm{tr}\left(\sigma U\sigma U^{†}\right)=\mathrm{tr}(|\psi \rangle \langle \psi |U|\psi \rangle \langle \psi |U^{†})=\langle \psi |U|\psi \rangle \langle \psi |U^{†}|\psi \rangle$, because the factors under the trace are cyclically permutable and 〈ψ|U|ψ〉 is a c-number.

Hamiltonian (9) is diagonal in the representation of the eigenfunctions |n〉; consequently, the evolution operator U(t)≡exp(−iHt) is also diagonal, with diagonal elements equal to its eigenvalues $\lambda_{n}\equiv \exp\left(-i\;\varepsilon_{n}t\right)=\exp\left[i\;(nx-n^{2})t\right]$. Applying the spectral theorem, we write the evolution operator as $U=\sum_{i}\lambda_{i}\left|i\rangle \langle i\right|$. Therefore, $\langle \psi |U|\psi \rangle \equiv \sum_{i}\lambda_{i}\langle \psi |i\rangle \langle i|\psi \rangle$ and

(16) $$\mathrm{tr}\left(\sigma U\sigma U^{†}\right)=\sum_{nm}\lambda_{n}\lambda_{m}^{*}\langle \psi |n\rangle \langle n|\psi \rangle \langle \psi |m\rangle \langle m|\psi \rangle .$$

Next, as ψ is the superposition of equally populated basis states, we find that $\langle \psi |n\rangle \langle n|\psi \rangle =\sum_{km}c_{k}^{*}c_{m}\langle k|n\rangle \langle n|m\rangle =c_{n}^{*}c_{n}=1/N$, and

(17) $$\mathrm{tr}\left(\sigma U\sigma U^{†}\right)=\frac{1}{N^{2}}\sum_{nm}e^{i\;\alpha_{nm}t}=\frac{1}{N^{2}}\sum_{nm}\cos\left(\alpha_{nm}t\right),\;\;\alpha_{nm}\equiv \left(n-m\right)x-\left(n^{2}-m^{2}\right),$$

where the imaginary terms of the sum are eliminated. They contribute zero, since for each term with indices nm there is a term with indices mn, while $\alpha_{nm}$ is antisymmetric under permutation n↔m.

Note that if the initial state were completely mixed, $\sigma =\bar{\rho }$, then the matrix $U\sigma U^{†}$, containing the possible dependence on the MF, see (15), as can be easily derived, would not depend on the MF. This means that in our model, as in the RPM where the initial state is usually singlet, it is essential that the initial state possesses purity, i.e., that $\mathrm{tr}(\sigma^{2})>1/N$. The initial state of the just-released rotator is pure.

Second, we need to find $\mathrm{tr}(\sigma \bar{\rho })$ in (15). Assuming, as before, $\bar{\rho }=\frac{1}{N}\sum_{k}\left|k\rangle \langle k\right|$, we obtain:

(18) $$\mathrm{tr}\left(\sigma \bar{\rho }\right)=\mathrm{tr}(|\psi \rangle \langle \psi |\frac{1}{N}\sum_{k}|k\rangle \langle k|)=\frac{1}{N}.$$

**Figure 2:**
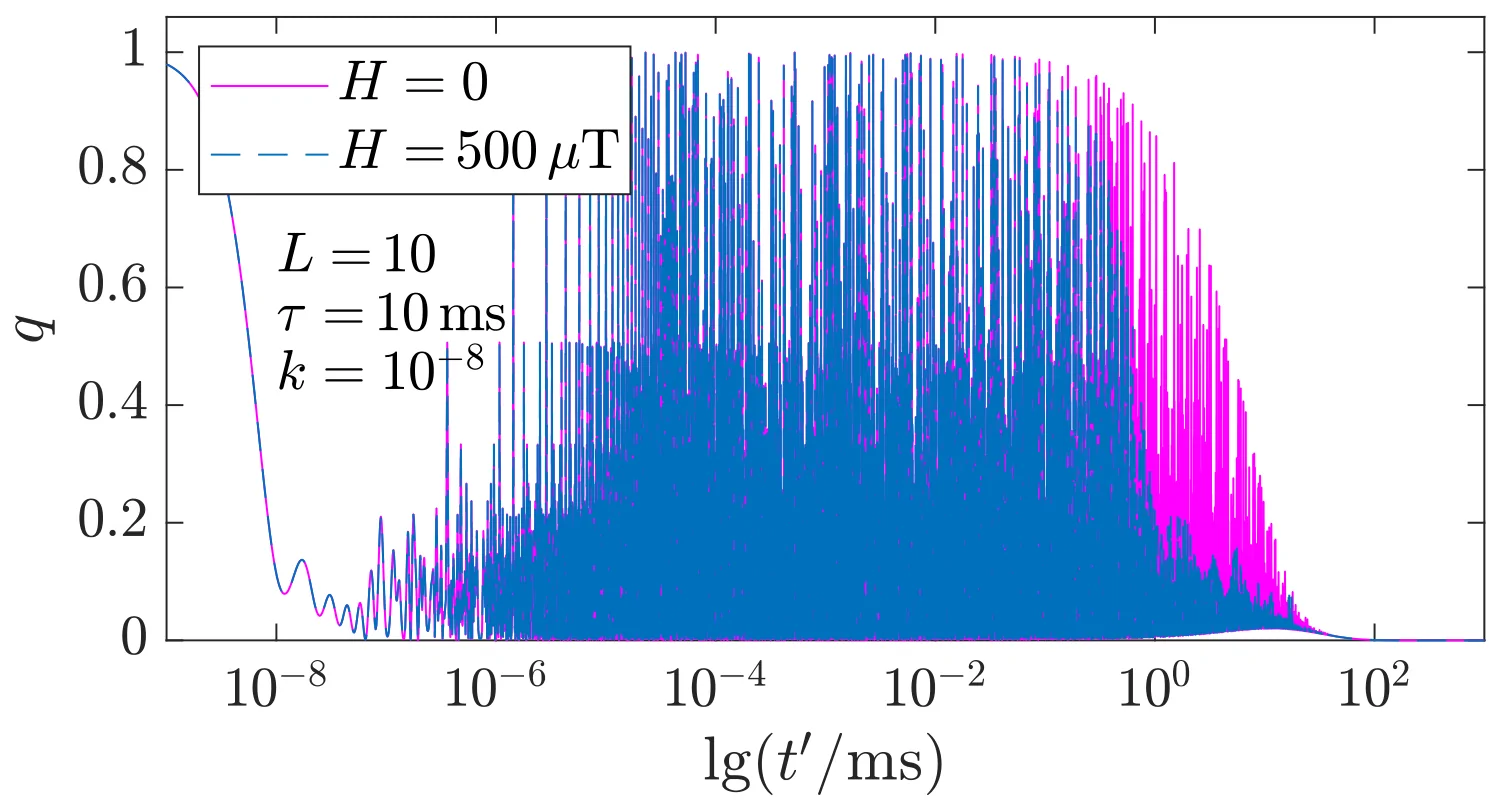
The time dependence of the population *q* of the state from which the biopolymerization error reaction occurs at different MFs. The values of parameters *L*, τ, and *k* are chosen for the best graphical representation of the processes. The difference in time scales between fast and slow dynamics reaches many orders of magnitude; therefore, the graph, built from several hundred points, is partly chaotic. The right tail of the functions is determined by the total rate of kinetics and decoherence.

The time dependence of the population *q*, after substituting relations (17) and (18) into (15) and converting to physical variables, is shown in Figure 2. The absence of an MF increases the time the rotator spends in the reaction-capable state, leading to an increase in the probability of an erroneous biochemical reaction.

Rapid oscillations are visible against the background of slow changes caused by the action of the MF, chemical kinetics, and relaxation processes. The rapid oscillations are a consequence of the nanoscopic nature of the rotator. As its mass increases, the gap between adjacent quantum levels decreases, and at a given temperature, many states become populated. The number of oscillation frequencies grows quadratically, which, superimposing on each other, leads to quasi-chaoticity of the small-scale dynamics. The time scales of the fast and slow dynamics differ by 8–9 orders of magnitude, making finding *q* by *numerical* solution of (2) inefficient. However, the rapid oscillations are averaged out by the subsequent time integration in the analytical estimation of the magnetic effect.

Now we are prepared to find the MF dependence of the averaged error probability. To connect with experimentally measurable quantities, it is necessary to integrate *q* over an interval which, due to the exponential decay of the function, see Figure 2, can be set to infinity, $F\equiv \int_{0}^{\infty }q\left(t\right)dt$. The population *q* in (15) consists of two terms. Therefore, *F* will also be the sum of two terms, $F=F_{1}+F_{2}$. Let us first compute the first integral, which, as follows from (6), (15), (17), and (18), is

(19) $$F_{1}\equiv \frac{1}{N^{2}}\sum_{nm}\int_{0}^{\infty }e^{-jt}\cos\left[\left(\alpha_{nm}-j\right)t\right]\;dt=\frac{1}{jN^{2}}\sum_{nm}\frac{1}{1+\alpha_{nm}^{2}/j^{2}},$$

where j≡k+g.

Next, among the terms in Equation (19), those with m≠±n satisfy $\alpha_{nm}\gg j$ and can therefore be neglected. For the terms with m=±n, one readily derives the relation

$$F_{1}\;jN^{2}=-1+N+\sum_{n}\frac{1}{1+4x^{2}n^{2}/j^{2}}+O\left(j^{4}\right).$$

Integration of the second term in (15) using (6) and (18) gives $F_{2}=\left(1/k-1/j\right)/N$. The next simplification is related to the fact that N≡2L+1≫1. Then, unity in $F_{1}\;jN^{2}$ can be neglected, *N* can be replaced by 2L, and the summation can be replaced by integration. Calculation of $F\equiv F_{1}+F_{2}$ yields

(20) $$F\left(x\right)\propto 1+\frac{k}{2Lx}\;\arctan\left(\frac{2Lx}{k+g}\right),$$

where we omit a common factor since it cancels in the subsequent calculation of the *relative* magnetic effect. The MF dependence of *M* appears in the next section.

As can now be seen, due to the properties of arctan(z)/z, the effect arises when Lx∼k+g. In physical variables (8), this gives, as expected, the same observability criterion (14). The relative magnetic effect tends toward zero both as k→0 and as k→∞. For significant values of *M*, the presence of chemical kinetics that is neither too fast nor too slow is necessary, as in the RPM. Furthermore, F(x)=F(−x), meaning that the magnetic effect is invariant under MF reversal, as is the case for the RPM.

### 2.4. Gyromagnetic Ratio of the Asp Residue

To obtain numerical estimates, we consider a specific example of a molecular rotator—an amino acid residue. Amino acids serve as monomeric units of protein polymers and are also present in the cytoplasm in free form. In the active site of the ribosome (the biopolymerization enzyme), an amino acid molecule covalently binds to the growing polypeptide chain.

As noted above, there are reasons to think that during ribosomal translation, a short time interval occurs in which the molecular fragment $H-C_{\alpha }-R$ in

$$\begin{array}{c} \mathrm{tRNA}\\ |\\ H-C_{\alpha }-R\\ |\\ \mathrm{protein} \end{array}$$

resides in a relatively free cavity within the enzyme’s active site, retaining the covalent bonds of C_α_ to both the tRNA and the growing protein chain, and can rotate about the axis defined by these two bonds. We assume that this amino acid residue is an integral dynamic unit and possesses one degree of freedom, the polar angle φ, which significantly simplifies the analysis.

The gyromagnetic ratio of the electron spin is $\gamma_{e}=\mu_{e}/\left(\frac{1}{2}\hbar \right)=g_{e}\mu_{B}/\hbar$, where $g_{e}\approx 2.0023$ is the electron g-factor, and $\mu_{B}=e\hbar /\left(2m_{e}c\right)$ is the Bohr magneton. Hence, $\gamma_{e}=g_{e}\;e/\left(2m_{e}c\right)\approx 1.76\times 10^{7}$ rad G^−1^ s^−1^. For the orbital motion of an electron, its gyromagnetic ratio is $\gamma_{\mathrm{or}}=e/\left(2m_{e}c\right)\approx 8.79\times 10^{6}$ rad G^−1^ s^−1^, where *c* is the speed of light in vacuum. This value is about two times smaller than the spin gyromagnetic ratio.

Let us estimate γ of an amino acid residue, assuming that various atoms and regions of charge density within the molecule perform rotations (or orbital motions), thereby creating the total mechanical and magnetic moments of the molecule. The gyromagnetic factor γ of the rotational motion of a system of point masses $m_{i}$ with charges $q_{i}$ is obtained from the gyromagnetic ratio of the orbital motion of an electron by the replacements $m_{e}\to I$ and e→Q, i.e., γ≡Q/2Ic, where $I\equiv \sum_{i}m_{i}r_{i}^{2}$ and $Q\equiv \sum_{i}q_{i}r_{i}^{2}$ are the moment of inertia of the masses and a measure of the charge distribution of the molecule; $r_{i}$ is the distance from the center of the point mass $m_{i}$ and charge $q_{i}$ to the axis of rotation [2] (p. 388).

Amino acids with a dipole moment along the side chain clearly have the largest gyromagnetic ratios. In glutamic and aspartic acids, carboxyl groups create a significant dipole moment. Methionine and cysteine contain sulfur-containing groups with high bond polarity. Lysine and arginine have long aliphatic chains with charged groups at the end.

Note that no exact value of γ exists for amino acid residues. The value of γ is determined by the distances of the atoms from the axis of rotation and by their partial charges, but both of these can vary depending on the specific amino acid sequence and conformational state. The estimates given below are approximate; it is plausible that the spread could reach 10–20%. For Asp and Glu, the gyromagnetic ratio was 120 and 110, respectively. Other amino acids, according to preliminary estimates, have γ in the range from 60 to 10.

The maximum gyromagnetic ratio is for the side chain of deprotonated aspartic acid, Asp, due to the large partial charge of the carboxyl oxygens, about 1.5 *e*, at a distance of about 0.32 nm from the axis of rotation. Estimation gives γ≈120 rad G^−1^s^−1^, which is $10^{5}$ times smaller than $\gamma_{e}$. However, the decoherence time τ of the rotational state of a similar fragment has been estimated to be 1–100 ms [8], which is approximately six orders of magnitude greater than the decoherence time of magnetosensitive electron spins in proteins, 3–30 ns according to [18]. Therefore, the product γτ, which determines the necessary minimum MF (5), can be even larger than that for the electron in a radical pair.

The deprotonated Asp residue in the geoMF rotates with an angular velocity of $\gamma H_{\mathrm{geo}}\approx 60$ rad/s (about 3 deg/ms). Over the rotational decoherence time, its angular displacement reaches several degrees—a significant value given the protein’s structural geometry and folding times. This displacement is neither negligible nor large enough to assume a uniform angular distribution. If the displacement is comparable to the width of the rotator’s quantum needle (Figure 1), then the absence of displacement would cause a biopolymerization error.

The magnetic RPM effect (including kinetics and spin decoherence) is defined in [18] to be zero in the absence of an MF and to increase with increasing MF. For the purpose of comparing the quantum rotator effect with the RPM effect, the magnetic effect is defined here in the same way:

(21) $$M\left(x\right)\equiv 1-\frac{F(x)}{F(0)}.$$

**Figure 3:**
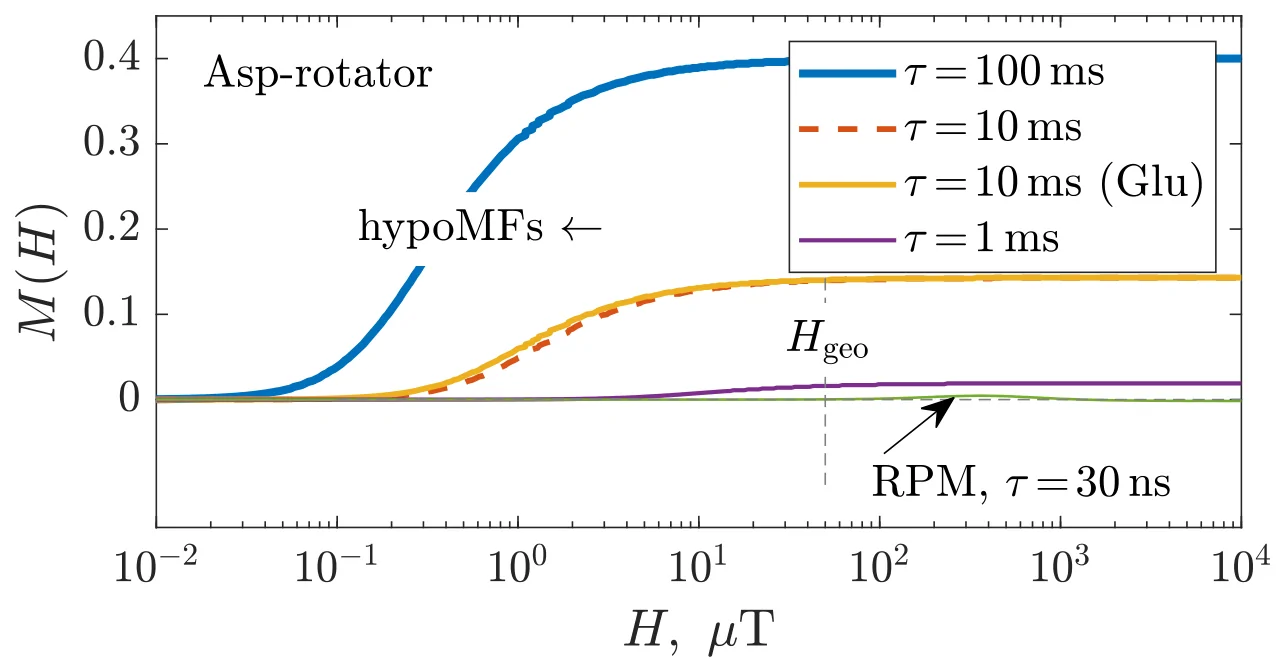
Dependence of the magnetic effect on the MF for a rotator simulating an Asp residue. The calculation follows (21) and subsequent conversion to physical variables (8) with a chemical kinetics rate κ∼20 s^−1^ and various quantum decoherence times τ. One of the curves is calculated for the Glu-rotator. For comparison, the analogous RPM MF dependence is shown for a typical RPM kinetics rate of $10^{6}$ s^−1^ and the largest τ=30 ns from the plausible interval of 3–30 ns [18].

The dependence of the magnetic effect on the MF is shown in Figure 3 for parameter values characteristic of Asp, with one curve corresponding to Glu. The amplitude of the effect increases with the rotational decoherence time τ, while the position of the inflection region shifts to a lower MF. The MF corresponding to the inflection region can be 1–2 orders of magnitude below the geoMF. The synthesis (elongation) rate of the protein chain is about 10–30 amino acids per second [31,32]. Accordingly, a value of κ=20 s^−1^ (corresponding to $k∼10^{-8}$) was used in the calculations of *M*.

## 3. Discussion

Microscopic magnetic moments are the first to interact with the MF. In the RPM, these are the spin magnetic moments of electrons. In the quantum rotator mechanism, they are the magnetic moments of electrons moving in orbits. In both cases, the energy of a magnetic moment is many orders of magnitude smaller than the thermal energy kT. On this basis, the well-known “kT problem” asserts the impossibility of magnetic effects in biology. However, today the kT problem is no longer relevant. It is inapplicable to either the RPM or the quantum rotator mechanism. In both theories, the magnetic response develops before thermal equilibrium is reached. In both theories, the thermal decoherence time τ accounts phenomenologically for the interaction with the thermostat.

The vector model of spins in the RPM considers the relative orientation of two spins, whereas in the quantum rotator scenario, the dynamics of the rotator’s magnetic moment are considered relative to its immediate environment [33]. In the early theory of the magnetosensitive rotator [8], which explained nonspecific magnetic biological effects, only the interference of the first few quantum states of an amino acid residue was considered—i.e., the case of small *L*. Therefore, the possibility of enhancing sensitivity to the MF through the interference of a large number of eigenstates remained unexplored. Furthermore, in that work, the magnetic effect, or the probability of a chemical reaction of the rotating molecular group, was attributed to the rotator being suspended at an arbitrary angular position.

In the present work, the scenario involving nanoscopic rotators in the biological response to a change in a weak MF is significantly advanced. We propose that during biological evolution, some processes with repeating catalytic cycles—in particular, ribosomal protein synthesis—have adapted to the presence of the geoMF. The geoMF rotates the rotator by several degrees during the thermalization time after its release. This rotation is the norm, ensuring optimal protein synthesis and proper folding. If this does not occur, for example in the absence of an MF, then amino-acid side chains remain longer in non-optimal positions. In the quantum rotator mechanism, the geoMF protects the organism from unfavorable effects, presumably owing to evolutionary adaptation, whereas a significant reduction in the MF to the level of a hypoMF leads to an increase in biopolymerization errors and associated disturbances. Some experimental biochemical evidence that the mitochondrion supporting ribosomal synthesis may be a target of MF in vivo is provided in [34].

From the perspective of applicability in magnetobiology, the quantum rotator mechanism contrasts sharply with the RPM. The magnetic effect already arises in an MF on the order of 1 μT (Figure 3), rather than in an MF on the order of 1 mT, as in the RPM. With decreasing decoherence time, the amplitude of the magnetic effect decreases almost linearly with τ, rather than as $\tau^{3}$, as in the RPM. This makes the effect under the quantum rotator mechanism, in a certain sense, weakly dependent on τ, since even with fast decoherence (τ=3 ms), the effect retains a magnitude of 5% that is convincing for magnetobiology. The RPM, with plausible spin decoherence times of 3–30 ns, yields a hypoMF effect of less than 0.1% [18].

Note that, as Figure 3 shows, a strong MF produces an effect almost identical to that in the geoMF. A strong MF induces rapid rotation of the probability density, to which the slow biochemical reaction does not respond.

Interestingly, temperature in this scenario not only suppresses the effect via decoherence but also gates its very existence: without the thermal population of the initial rotator states, the interference pattern would lack the sharp spike whose field-induced motion defines the magnetic effect.

The fact that the quantum rotator mechanism relies on quantum rotations does not require the rotator to be a rotor, i.e., to rotate freely through a full circle or beyond. As shown above, only small angular displacements from the rotator’s initial position after its release are sufficient for the effect. Hence, the magnetic effect can manifest even when rotations of amino acid residues are limited by steric factors or take place in complex rotational potentials.

It is important that a fraction of the MF sensors in the form of quantum rotators is located on large rotating molecular aggregates, for example, on DNA strands [33]. In the absence of macro-rotations, the minimum MF is given by (14) as 1/Lγτ (κ≪τ). In the presence of rotations, however, the response becomes a bell-shaped function of width 2/Lγτ, shifted relative to zero MF by v/γ, where *v* is the rotation speed. Consequently, the effect lacks symmetry under MF reversal, which allows the rotator mechanism to be distinguished from the RPM.

It is easy to estimate that the rotation speed of a molecular aggregate carrying a nanoscopic quantum rotator would need to be about 1–10 rps to shift its response into the geoMF region. Such rotations are common in biophysical structures within organisms. It is known that the rotation speed of ribosome parts (translation) is approximately 0.5 rps. The relative rotation speed of DNA and RNA polymerases (transcription) is about 5 rps. The rotation speed of helicases (DNA unwinding) and topoisomerase II (removing supercoiling) ranges from units to tens of rps. Myosins rotate around the actin filament at 1.5–2.5 rps, and dynein rotates at 1–4 rps. These rotations [35,36,37] are not an epiphenomenon but a fundamental property of molecular machines.

Thus, the consideration of intrinsic rotations of enzymes carrying molecular rotators provides high sensitivity to MF variations against the background of the geoMF. This makes it possible to interpret animal magnetic navigation, i.e., their use of the Earth’s magnetic relief (variations at the level of tens of nT) in thousands-of-kilometer migrations to seasonal habitats. It could also explain the intriguing correlation between geomagnetic disturbances and certain biological processes under laboratory conditions [38]. Evidence for a causal relationship in humans has long remained inconclusive due to the disparity between the amplitudes of geomagnetic disturbances and the diversity of MFs in which humans are constantly immersed. However, systems have now emerged that are capable of recording and reproducing a magnetic storm in the laboratory, yielding interesting results on the direct effect of artificial storms on humans [39,40]. In light of the quantum rotator mechanism presented above, these results do not appear to be contradictory.

### 3.1. Comparison of Mechanisms in Magnetobiology Based on Quantum Rotations and Radical Pairs

An important merit of the RPM in explaining animal magnetic orientation is the dependence of the effect on factors promoting radical pair formation, particularly optical radiation of a specific spectral range. This dependence is considered direct evidence for the involvement of radical pairs in magnetoreception. Notably, molecular rotators appear in enzyme active sites where electron transfer processes occur—conditions conducive to radical pair formation. Therefore, the involvement of the RPM in the process generating quantum rotators susceptible to MF is plausible, and the dependence of the quantum rotator mechanism effect on illumination is not excluded. In other words, the observed influence of radiation of a specific spectral range on magnetoreception in some migrants does not rule out a definitive role for quantum rotators in animal magnetic navigation. The authors of experiments with differently illuminated plants in an MF [41,42] interpreted their results based on the dynamics of a single molecular magnetic moment, doubting the RPM-based explanation.

The effect magnitude depends on the MF orientation relative to the rotator’s rotation axis. An MF perpendicular to the axis has no effect because the charge rotation contour encloses no MF lines. The projection onto the axis follows a cosine law. Therefore, if quantum rotators participate in signaling protein synthesis and their carrier enzymes are uniformly oriented, a directional dependence of the biological response on MF orientation, similar to that in RPM, cannot be ruled out.

Above, we considered an example involving an amino acid residue appearing in the active site of the ribosome during translation. One might also suppose that during protein folding, virtual regions devoid of water molecules could form, allowing an amino acid residue to rotate in the MF. This would imply a direct action of the MF on the folding of some proteins. Furthermore, there is currently no apparent reason why similar processes could not occur in DNA–RNA biopolymerization enzymes, particularly in those of repair systems. The quantum rotator mechanism currently lacks experimental evidence pointing to any specific MF target. The quantum rotator may be embedded in a biopolymerization process, but the exact nature of this process remains unclear.

Table 1 summarizes the main characteristics of the RPM and the quantum rotator mechanism. These mechanisms are considered in relation to magnetobiology. Both mechanisms share a common foundation: the quantum dynamics of electronic magnetic moments—two spins for RPM and an orbital moment for the rotator mechanism. In both cases, dynamics are described using the Liouville–von Neumann equation. However, the outcomes differ.

As can be seen from Table 1, the two mechanisms share many common properties. Their main difference lies in the nature of the magnetic moment interacting with the MF: the spin magnetic moment in the RPM versus the orbital magnetic moment in the rotator mechanism. In both mechanisms, the quantum decoherence time is a key factor determining the magnitude of possible magnetic effects. The effect magnitude in the rotator mechanism is larger by approximately three orders of magnitude. Even if its decoherence time were two to three orders of magnitude smaller than the current theoretical estimate—an unlikely scenario—this mechanism would still represent an attractive conceptual alternative to the RPM.

### 3.2. Distinguishing Between the RPM and the Quantum Rotator Mechanism

Can the two mechanisms, the rotator mechanism presented above and the widely discussed RPM, be distinguished experimentally? Below, we consider various approaches:

- *Weak-Field Dependence of M(H):* In the RPM, the effect in weak fields is well known to be proportional to $H^{2}$ when the reference point is a “zero” MF. If, in the rotator mechanism, the reference point is taken as H=0 rather than $H_{\mathrm{geo}}$, then at low fields (up to the inflection point) the effect is also proportional to $H^{2}$. Thus, this method cannot distinguish between the two mechanisms.
- *Response to MF Reversal:* In the rotator mechanism, reversing the MF changes the direction of quantum rotation. Because the magnetic effect is associated with the quantum needle being suspended at its initial position, the direction in which the needle shifts is evidently irrelevant. Consequently, the magnetic effect is invariant under MF reversal. Thus, this method also cannot experimentally distinguish the quantum rotator mechanism from the RPM.
- *Response to Rotation of the Sample Containing the Cell Culture:* In the RPM, such a procedure does not affect the magnitude of the effect, as it depends (in the vector model) on the *relative* orientation of the radical pair spins. In the rotator mechanism, external rotation of the sample at 1–10 Hz would likely disrupt the hypoMF effect. However, this requires further quantum-mechanical evaluation and will be addressed in a separate article.
- *Position of the Inflection Point on the M(H) Curve:* This seems to be the most reliable way to distinguish between the two mechanisms, as their inflection point values differ by two to three orders of magnitude.
- *Behavior of M(H) near the geoMF:* Since the RPM exhibits no distinctive behavior near the geoMF, the response *magnitude*, i.e., absolute change, upon changing the MF from the geoMF to $\pm H_{\mathrm{geo}}/2$ would be almost symmetric. In the rotator mechanism, by contrast, the same MF change would produce substantially different asymmetric responses, Figure 3.
- *Response to a Resonant MF:* Obviously, the normal quantum rotation of a nanorotator in the geoMF can be disrupted not only by eliminating the geoMF but also by applying an additional alternating MF. This would destroy the finely tuned interference of hundreds of rotational levels that is necessary for the magnetic biochemical effect to occur. For Asp, the characteristic frequency ω≡ℏ/2I, i.e., the transition frequency between the zeroth and first quantum levels of the rotator, is about 1 GHz and is independent of the MF. In the RPM, the Zeeman spin sublevels in the geoMF fall into the MHz range. Adding an MF of such frequencies in an experiment would be a possible way to distinguish between the two mechanisms, were it not for two circumstances. First, the application of MHz and GHz MFs can induce, and most likely does induce, significant electrical phenomena in biological tissue, particularly the absorption of the RF/microwave field energy. This would complicate the interpretation of the experimental results. Second, unlike in spin systems, the application of an additional alternating perpendicular MF does not induce changes, as noted above. Modulation of a static MF parallel to the rotator axis also does not induce changes, since a unidirectional MF does not induce transitions in states that are eigenstates with respect to this. Between the Zeeman sublevels ±x of the first rotator level in a 50 μT geoMF, the frequency is approximately $\gamma H_{\mathrm{geo}}/\pi ∼19$ Hz. However, inducing transitions between them is also impossible for the same reason. Thus, this scenario for distinguishing between the mechanisms also fails.
- *Response to a Pulsed MF:* A suitable approach involves using pulsed MFs with smoothed fronts to avoid inducing significant electric fields. For example, pulses of width $\Delta t^{\prime }=1$ ms and amplitude $2\pi /\gamma \Delta t^{\prime }\approx 5$ mT, added to a zero MF, would rapidly rotate the rotator’s probability density interference pattern by a full angle of 2π. Consequently, the time-averaged position of the quantum needle would remain nearly unchanged, and the hypomagnetic effect would persist regardless of pulse frequency. The effect would disappear if the rotation angle deviated significantly from 2π upon varying the pulse amplitude. In the RPM, by contrast, this scenario would destroy the hypomagnetic effect irrespective of pulse shape fine-tuning, as the RPM effect develops on a tens-of-nanoseconds timescale. Here, the effect would be equivalent to that produced by the average MF of the pulse train, i.e., it would depend on pulse frequency for a fixed pulse shape. However, because the pulse amplitude cosinusoidally depends on the MF direction, this discrimination method is applicable only to uniformly oriented enzyme arrays. Cell cultures of certain plants may possess such a property [42].
- *Biochemical Method of Discrimination:* Large γ values favor the emergence of a significant quantum rotator effect. Among the amino acid residues that are potential candidates for quantum nanorotators, deprotonated Asp and Glu stand out due to their large absolute values of γ (120 and 110, respectively). Consequently, when searching for the molecular target of MFs in organisms, it would be prudent to focus on the biosynthesis of proteins enriched in these and related amino acids.

In magnetobiology, the RPM has clearly focused on radical pairs in cryptochromes as a likely MF target, at least for bird magnetoreception. Thus, a specific link between spin RPM dynamics and a biochemical process in organisms has been proposed based on experimental evidence. In contrast, the quantum rotator mechanism currently lacks an MF target identified with comparable confidence. Quantum rotators may be embedded in biopolymerization, but the specific process involved remains unclear.

In our view, the simultaneous assessment of both the concentration and functional activity of a given protein could reveal alterations in the relative abundance of nonfunctional molecules, or a slowdown in translation, in the hypoMF compared with the geoMF.

### 3.3. Possible Influence on the Functionality of Certain Proteins

Because an MF response is found in taxa [5] that diverged hundreds of millions of years ago, it is likely that their common ancestor already possessed this ability and that it has been conserved. This is not about magnetic nanoparticles, as their occurrence in organisms is much rarer than the magnetic response itself.

At first glance, within the molecular rotator framework, it would be reasonable to hypothesize the existence of some protein common to all organisms that is enriched in Asp. For example, the protein Aspolin contains up to 96% Asp residues [43], but it has been found only in fish. Other amino acid residues with a relatively small size and a high gyromagnetic ratio could also be of interest. These include serine (γ∼30 rad G^−1^s^−1^) and threonine (γ∼20 rad G^−1^s^−1^). In all eukaryotic organisms, from humans and plants to fungi, there are proteins with elevated serine content, reaching up to 20% in some regions.

Universal proteins rich in amino acid residues with elevated γ appear to be abundant. In the RPM, the primary candidates for the molecular MF target are cryptochromes, whose main role is blue light perception and, consequently, circadian rhythm regulation. However, cryptochromes are not as universal as, say, SR proteins (serine/arginine-rich proteins), which act as splicing regulators [44]. Thus, it seems plausible that if a universal molecular mechanism of magnetic response exists across all organisms, it is not tied to any specific amino acid but rather to the biochemical role of small rotations—potentially controlled by the MF—of amino acid residues with a large gyromagnetic ratio within enzyme active sites during biopolymerization.

Interestingly, when an external toxic factor, poly-PR, reduced the function of one SR protein (SRSF7), it impaired the regenerative capacity of nerve axons, resulting in neuronal dysfunction [45]. It is reasonable to suppose that a reduction in SR protein function caused by a different external factor—namely, an MF—could similarly lead to neuronal effects.

The universality of proteins like SR proteins, on the one hand, and the dependence of MF-induced rotations on a wide range of physical, biochemical, and physiological conditions, on the other, render the occurrence of a magnetic response in organisms largely accidental. Such molecular-level randomness seems to account for the notoriously poor reproducibility of magnetobiological effects [46]. In this context, it is worth noting that if the rotator mechanism holds, the absence of a response to an MF stronger than the geoMF does not imply that a response to a hypoMF will also be absent. The “common sense” intuition that a stronger stimulus produces a stronger response fails here.

To date, there have been virtually no publications on the direct effect of a hypoMF on biopolymerization in a cell-free in vitro system, probably because of the difficulty of isolating individual biochemical branches in typical biological processes. Indirect effects of a hypoMF on gene expression have been reported [47,48,49,50,51,52,53,54,55,56]. Effects on protein synthesis have been described [57,58,59,60,61]. Processes involving DNA (chromatin conformation, repair) are discussed in [62,63,64,65].

## 4. Methods

The estimates of gyromagnetic ratio γ were obtained as follows. The distances from C_α_ to the side-chain atoms of deprotonated Asp and Glu were determined in PyMOL 3.1.1, and the partial charges of the atoms of these side chains were obtained using the web application “PDBCharges” [66]. The Asp residues were taken as part of the miniproteins Chignolin (PDB ID: 1UAO) and Trp-cage (1L2Y), and the Glu residues as part of the miniproteins Chignolin and Contryphan-Vc1 (2N24). The charge and distance values averaged over the pair of proteins were used to calculate γ. The resulting values were rounded to two significant figures.

All calculations and visualizations were performed using MATLAB R2024a.

## 5. Conclusions

We propose a mechanism for the hypoMF effect in organisms based on the quantum behavior of a nanoscopic molecular rotator. The magnetic effects were calculated by applying a known solution of the Liouville–von Neumann equation—incorporating chemical kinetics and phenomenological decoherence—to a molecular rotator of about 0.5 nm in size. In this mechanism, the rotator’s quantum dynamics models the rotation of an amino acid residue within the active site of a biosynthesizing enzyme. Thus, the reaction rate is determined not by spin-based prohibitions, as in the RPM, but by the suppression of quantum rotation.

We demonstrate that magnetic effects reaching up to several tens of percent arise from modulation of the rotator’s quantum interference pattern in very weak MFs, and that the effect is relatively robust to decoherence. Temperature, as is typical, promotes decoherence, but it also enables the very existence of magnetic effects by facilitating the emergence of a finely structured, magnetically responsive interference pattern prior to the onset of decoherence. Under realistic parameter values, the rotator’s probability density rotates by a few degrees in the geoMF, a finding that may help interpret various magnetobiological phenomena.

The quantum rotator mechanism implies that evolutionary adaptation has allowed organisms to harness the geoMF, rendering biosynthesis nearly error-free. When the MF is reduced to hypoMF levels, biosynthetic errors increase, which could manifest at the organismal level.

The main strength of the RPM—which is actively discussed in magnetobiology—lies in its firm grounding in spin chemistry. Its principal weakness, however, is its low sensitivity to MFs, stemming from rapid spin decoherence. As a result, explaining animal magnetic orientation, navigation, and the biological effects of magnetic storms has long been riddled with inconsistencies. The quantum rotator mechanism circumvents this obstacle, at least in principle.

The high responsivity of the rotator mechanism stems from the fact that it operates beyond the standard quantum limit (5). This limit is rooted in the energy–time uncertainty relation, whereas in the quantum rotator mechanism, the MF acts by influencing the phases of wave functions rather than the energy of the magnetic moment. The fundamental physical limit transforms into (14) when a typically biological process such as biopolymerization is incorporated into the system. Here, the chemical reaction occurs only at a specific angular orientation of the rotator and is governed by the MF, which shifts the rotator’s position. If this holds true, then, metaphorically speaking, organisms learned to lift a physical constraint by modifying it, exploiting quantum interference at a transient yet crucial stage of a biological process.

The principal strength of the mechanism lies in its biophysically transparent explanation of the biological effects induced by very weak MFs. The quantum rotator mechanism is insensitive to fields substantially stronger than the geoMF; it simply does not respond to them. Thus, the RPM and the quantum rotator mechanism operate in distinct MF intensity ranges: the former above 1 mT, the latter below $H_{\mathrm{geo}}$.

A limitation of this mechanism—likely a temporary one—is its emerging character. At present, no experimental evidence supports the viability of the rotator mechanism, unless one counts the very effects that resist explanation by the RPM framework. It seems that the quantum rotator mechanism currently offers the only working interpretation of laboratory-observed biological effects of hypoMFs, as well as those of magnetic storms and animal magnetic navigation.

The molecular rotator is not merely a quantum-mechanical object but a nanoscopic system in which quantum effects can become observable under biological conditions. This is a case where quantum effects arise not despite biology, but because of it. The extreme responsiveness of nanorotator dynamics to parameter variations—particularly to the MF—may represent a previously unexplored resource in molecular biology, opening new perspectives for theoretical and experimental research.

## Figures and Tables

**Table 1:** Comparison of RPM in magnetobiology and quantum rotator mechanism.

PROPERTY	RPM	QRM
**General:**		
Origin	Spin chemistry	Biophysics
Status in magnetobiology	hypothesis	hypothesis
Experimental verification: confirming/refuting	yes/yes	no/no
Master equation	Liouville–von Neumann	Liouville–von Neumann
**Quantum state:**		
Primary degree of freedom	virtual (intermediate) spin	virtual (intermediate) orbital
Nature of magnetic moment	electron spin	spatial motion of rotator electrons
Quantum state of magnetic moment	metastable	metastable
Metastability due to	weak coupling to environment, angular momentum conservation law	The same as in RPM
Cause of metastable state formation	quantum tunneling of an electron leading to spatially separated radicals	release of the rotator from environmental bonds, leading to free rotation
Observability of virtual degrees of freedom	The relative number of degrees of freedom is insufficient for spectroscopic detection. MF is the main detection tool for the intermediate state	The same as in RPM
**Selectivity:**		
Selective reactivity consists in	failed reaction due to back-tunneling of an electron	failed reaction due to angular displacement of the rotator from state where a biochemical reaction occurs
Selective reaction occurs	despite thermalization and before it completes	due to thermalization and before it completes
Reason for selectivity	conservation of angular momentum	quantum rotation
Magnetic field	removes the radical pair from the state where back-tunneling is possible	removes the rotator from the state where a biochemical reaction is possible
Decoherence time	$\tau_{e}$, 3–30 ns	τ, 1–100 ms
Gyromagnetic ratio	$\gamma_{e}=1.76\times 10^{7}$ rad G^−1^s^−1^	γ≈120 rad G^−1^s^−1^ (Asp)
Main observability criterion	$H>1/(\gamma_{e}\tau_{e})∼1$ mT	H>1/(Lγτ)∼1 μT
**Challenges:**		
Plausibility of selective step	superexchange in cryptochromes—hypothesis	biopolymerization error in ribosomes—hypothesis
Photoactivation	required	not required
Response to MF reversal ±H: stationary case/rotating carrier	symmetric/symmetric	symmetric/asymmetric
Magnetic effect in geoMF	<0.1%	up to 40%
Proposed biophysical MF target	Cryptochrome CRY4	Ribosome
Animal magnetic navigation and geomagnetic storms	no interpretation	can be interpreted
RF/microwave biological effects	no interpretation	can be interpreted

## Data Availability

No new data were created or analyzed in this study. Data sharing is not applicable to this article.
